# Cardiac MR detects impaired myocardial perfusion reserve and left-ventricular hypertrophy in C57Bl/6 mice fed a high-fat diet

**DOI:** 10.1186/1532-429X-16-S1-O87

**Published:** 2014-01-16

**Authors:** Nivedita K Naresh, Xiao Chen, Rene J Roy, Brian H Annex, Frederick H Epstein

**Affiliations:** 1 Biomedical Engineering, University of Virginia, Charlottesville, Virginia, USA; 2 Radiology, University of Virginia, Charlottesville, Virginia, USA; 3 Cardiovascular Medicine, University of Virginia, Charlottesville, Virginia, USA

## Background

Ischemic heart disease is a major cause of mortality in the western world. Reduced myocardial perfusion reserve (MPR) is an independent predictor of cardiac mortality in patients with known or suspected coronary artery disease (CAD). An emerging concept is that impaired MPR can occur in the absence of obstructive CAD, especially in patient populations of women, diabetics and patients with the metabolic syndrome, where it is also predictive of adverse events. Mouse models can elucidate molecular mechanisms that underlie cardiovascular disease. We hypothesized that mice fed a high-fat diet (HFD) recapitulate the clinical scenario of impaired MPR without obstructive CAD in diabetic patients.

## Methods

C57Bl/6 mice fed a HFD for 18 (n = 7) and 24 weeks (n = 6) and age-matched C57Bl/6 mice fed a standard chow diet (Control, n = 6) were imaged at 7T. The HFD mice were selected from a larger group based on their glucose intolerance. Mice were anesthetized with 1.25% isoflurane and maintained at 36 ± 0.5°C during MRI. The MR protocol included multi-slice cine imaging to assess ejection fraction, left-ventricular (LV) mass, LV wall thickness, and LV volumes, cine DENSE imaging to quantify myocardial strain, and first-pass imaging at rest and with the vasodilator Regadenoson (0.1 μg/g body weight) to quantify MPR. A compressed-sensing accelerated dual-contrast saturation-recovery sequence was used to acquire first-pass Gd-enhanced images, and Fermi function deconvolution quantified perfusion and MPR. Histology of the aorta detected the presence or absence of systemic atherosclerosis, and myocardial capillary density was quantified.

## Results

**Figure 1:**
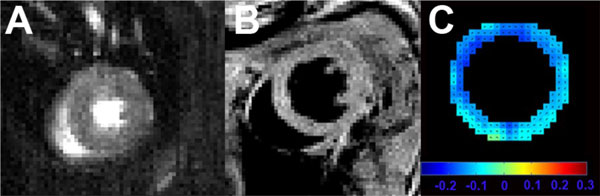
**Example first-pass gadolinium-enhanced image (A), cine image (B) and DENSE strain map (C) of the mouse heart**.

**Figure 2:**
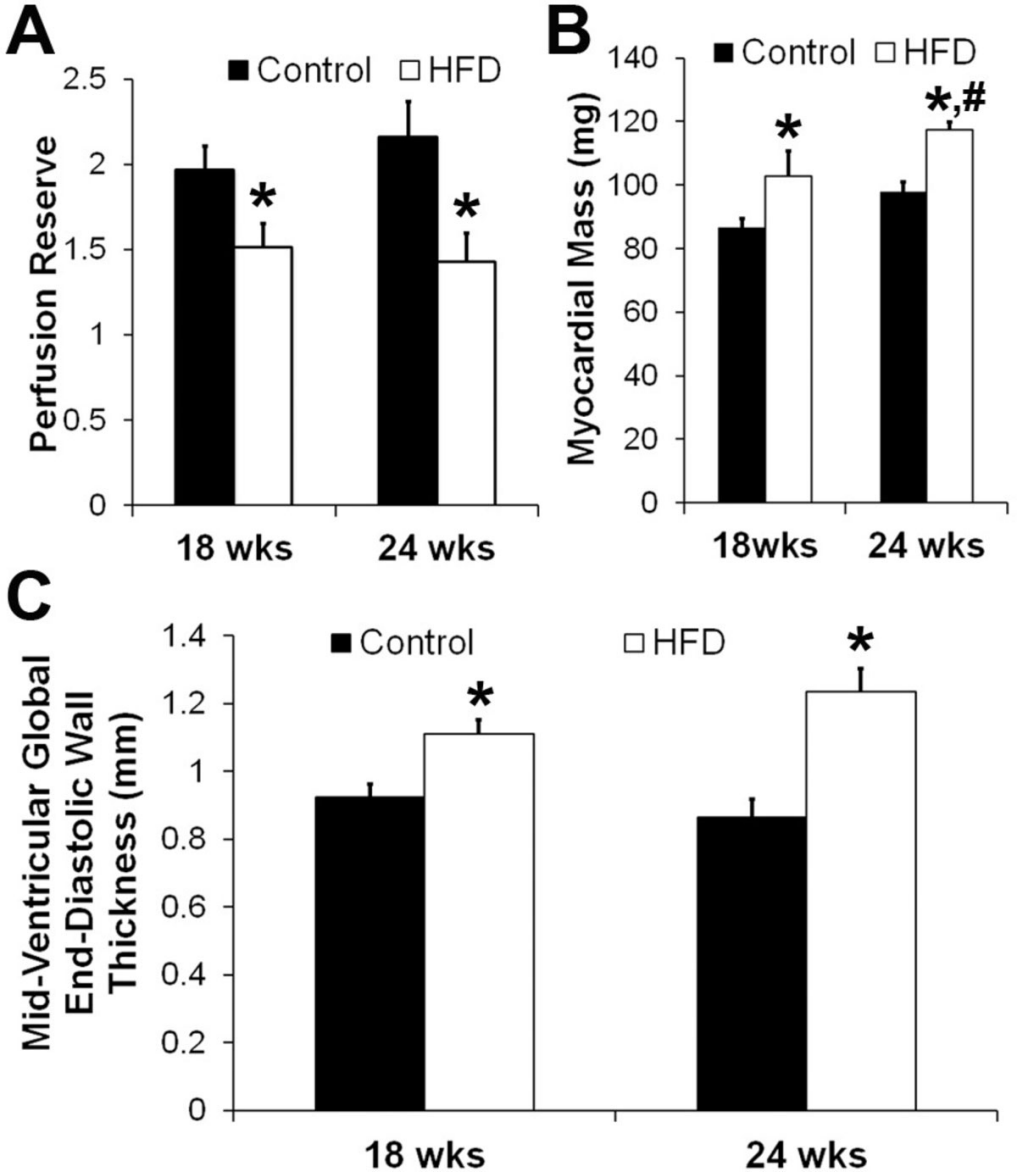
(A): In control mice, MPR was 2.0 ± 0.1 and 2.2 ± 0.2 at 18 wks and 24 wks respectively and it reduced to 1.5 ± 0.1 and 1.4 ± 0.2 in the HFD mice (*p < 0.05 vs. age-matched control). (B): LV mass was increased in HFD mice at 18 wks (*p < 0.05 vs. control) and it further increased at 24 wks (*p < 0.05 vs. control, #p < 0.05 vs. HFD at 18 wks). (C): Mid-ventricular end-diastolic wall thickness was increased in HFD mice at 18 and 24 wks after start of diet (*p < 0.05 vs. age-matched control).

HFD mice were obese relative to Control mice at 18 weeks of diet (49.4 ± 1.8 g vs. 29.5 ± 1.0 g, p < 0.01) and their body weight further increased at 24 weeks (55.2 ± 1.3 g vs. 30.9 ± 0.4 g, p < 0.01). Figure 1 shows examples of (A) first-pass perfusion MRI, (B) cine MRI, and (C) DENSE MRI from a mouse heart. MPR in HFD mice was reduced (Figure 2A, p < 0.05 vs. age-matched control). LV mass was increased in HFD mice at 18 weeks (p < 0.05 vs. Control) and it further increased at 24 weeks (p < 0.05 vs. Control, HFD at 18 weeks) (Figure 2B). LV wall thickness (Figure 2C) was increased in HFD mice (p < 0.05 vs. age-matched Control). There were no significant differences in myocardial strain, volume, ejection fraction and capillary density measurements between the two groups. Histology showed no aortic atherosclerosis in HFD or Control mice.

## Conclusions

Using cardiac MR, we showed that C57Bl/6 mice fed a HFD for 18-24 weeks with glucose intolerance have reduced MPR, increased LV mass, increased wall thickness and no aortic plaque. The HFD mouse model recapitulates early cardiovascular abnormalities of diabetic patients without obstructive CAD. Future studies using cardiac MR and gene-modified mice fed a HFD may shed light on key molecular mechanisms that underlie myocardial ischemia in the absence of obstructive CAD.

## Funding

This work was funded in part by AstraZeneca, NIH R01 EB001763 and US-Israel Binational Science Foundation grant 2011328.

